# A New Joint-Blade SENSE Reconstruction for Accelerated PROPELLER MRI

**DOI:** 10.1038/srep42602

**Published:** 2017-02-16

**Authors:** Mengye Lyu, Yilong Liu, Victor B. Xie, Yanqiu Feng, Hua Guo, Ed X. Wu

**Affiliations:** 1 Laboratory of Biomedical Imaging and Signal Processing, The University of Hong Kong, Pokfulam, Hong Kong SAR, China; 2 Department of Electrical and Electronic Engineering, The University of Hong Kong, Pokfulam, Hong Kong SAR, China; 3 School of Biomedical Engineering, Southern Medical University, Guangzhou, China; 4 Center for Biomedical Imaging Research, Department of Biomedical Engineering, Tsinghua University, Beijing, China

## Abstract

PROPELLER technique is widely used in MRI examinations for being motion insensitive, but it prolongs scan time and is restricted mainly to T2 contrast. Parallel imaging can accelerate PROPELLER and enable more flexible contrasts. Here, we propose a multi-step joint-blade (MJB) SENSE reconstruction to reduce the noise amplification in parallel imaging accelerated PROPELLER. MJB SENSE utilizes the fact that PROPELLER blades contain sharable information and blade-combined images can serve as regularization references. It consists of three steps. First, conventional blade-combined images are obtained using the conventional simple single-blade (SSB) SENSE, which reconstructs each blade separately. Second, the blade-combined images are employed as regularization for blade-wise noise reduction. Last, with virtual high-frequency data resampled from the previous step, all blades are jointly reconstructed to form the final images. Simulations were performed to evaluate the proposed MJB SENSE for noise reduction and motion correction. MJB SENSE was also applied to both T2-weighted and T1-weighted *in vivo* brain data. Compared to SSB SENSE, MJB SENSE greatly reduced the noise amplification at various acceleration factors, leading to increased image SNR in all simulation and *in vivo* experiments, including T1-weighted imaging with short echo trains. Furthermore, it preserved motion correction capability and was computationally efficient.

In periodically rotated overlapping parallel lines with enhanced reconstruction (PROPELLER) magnetic resonance imaging (MRI)^1^,^2^, multiple small Cartesian data sets, referred to as blades, are acquired with rotating phase encoding directions. A portion of the central k-space is repetitively sampled in all blades, such that every individual blade can provide a low-resolution image for motion correction. PROPELLER has been widely used for T2-weighted imaging in clinical examinations, where typically each blade is acquired by a single-shot fast spin echo. PROPELLER has also been explored for other applications such as diffusion weighted imaging^3^,^4^,^5^,^6^ and water-fat separation^7^,^8^,^9^. The main disadvantage of PROPELLER is the increased scan time. To alleviate this issue and facilitate accurate motion correction, PROPELLER is routinely acquired using long echo train length (ETL) (e.g., ETL = 30). This long ETL requirement confines PROPELLER to mainly T2-weighted contrast^10^, and can lead to high specific absorption ratio (SAR) and limit the number of slices acquired per repetition time (TR).

A number of studies^5^,^8^,^10^,^11^,^12^,^13^,^14^ have investigated parallel imaging acceleration on PROPELLER. Parallel imaging acceleration can widen blades, thus reducing scan time with fewer blades to cover the entire k-space. Alternatively, it can be used to reduce the ETL, leading to more flexible contrasts and reduced SAR^5^,^10^.

Nevertheless, noise remains to be one primary issue in accelerated PROPELLER, particularly at high accelerations. The noise level not only determines the image quality but also affects the motion correction accuracy. PROPELLER trajectory has the intrinsic potential of achieving low noise amplification. Different rotatory blades spread the aliasing into different directions, fully exploiting the coil sensitivity encoding capacities in the two dimensional plane, as similar to some non-Cartesian techniques^15^. Different blades, with distinct aliasing patterns, can provide complementary information and together provide more robust reconstruction.

A relatively straightforward reconstruction strategy has been commonly used for accelerated PROPELLER: each blade is separately reconstructed using parallel imaging reconstruction^16^,^17^, and then all blades are combined with motion correction^5^,^9^,^10^,^12^. We refer to such conventional strategy as simple single-blade (SSB). SSB strategy has been well demonstrated at relatively low accelerations. However, at higher accelerations, it may manifest its limitations in noise amplification. Since the blades are simply combined after their individual reconstruction, certain ill-conditioned blades can dramatically increase the noise amplification in the final images^12^. Due to the lack of information sharing among individual blades, the reconstruction of these ill-conditioned blades cannot be improved even if other relatively well-conditioned blades contain usable information. One way to overcome this problem is to jointly reconstruct all blades simultaneously, which may result in global optimization and suppress the noise. In addition, the blade-combined images as in the traditional PROPELLER reconstruction can serve as the regularization references to improve the reconstruction of individual blades.

Here, we propose a new multi-step joint-blade (MJB) SENSE^16^ reconstruction procedure, particularly suitable for highly accelerated PROPELLER. To reduce noise amplification, MJB SENSE utilizes all blades jointly, including joint-blade SENSE and regularization using the blade-combined images. The entire procedure consists of three steps. First, SSB SENSE is employed to provide blade-combined images. Second, using the blade-combined images as regularization references, individual blades are reconstructed again with reduced noise. Third, all acquired blades are widened virtually, with data resampled from the blade-combined images updated in the second step, and the widened blades are reconstructed using joint-blade SENSE to form the final images.

In this paper, the procedure of MJB SENSE is described first. The performance of MJB SENSE is evaluated and compared with SSB SENSE using numerically simulated phantom and brain data for noise reduction and motion correction, at various acceleration factors and noise levels. The proposed MJB SENSE is also applied to *in vivo* brain data, including both T1-weighted and T2-weighted contrasts.

## Methods

### Simple Single-Blade SENSE (SSB SENSE)

First, we briefly review simple single-blade SENSE^5^,^12^. Let *R* denote the acceleration factor, *N*_*c*_ the coil number, *N*_*b*_ the blade number, *W* the blade width, and *L* the number of data points per k-space line. First, the blades are transformed to image space by inverse 2D fast Fourier transform (FFT), resulting in *N*_*b*_ × *N*_*c*_ aliased blade images. Coil sensitivity maps (CSMs) are rotated to match the angle of each blade. Then, SENSE is performed within each single blade separately. The so-called single-blade SENSE equation in a blade *i* can be written as^16^:


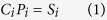


where *i* is the blade index, *P*_*i*_ = [*ρ*_1,*i*_*ρ*_2,*i*_ … *ρ*_*R*,*i*_]^*T*^ the pixels to be solved (*FOV*/*R* away from each other), *C*_*i*_ = [*C*_1,*i*_*C*_2,*i*_ … *C*_*R*,*i*_] the coil sensitivities on *P*_*i*_, with coil dimension implicitly included in each column vector, and 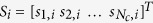 the signal intensities in the aliased blade images. Assuming *C*_*i*_ is known and noise prewhitening^18^ has been done, SSB SENSE can estimate *P*_*i*_ as:


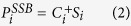


where 
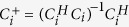
 is the Moore–Penrose pseudoinverse of *C*_*i*_^16^, and 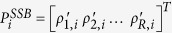 is the plain least-squares estimation of *P*_*i*_. By solving all single-blade equations individually, totally *N*_*b*_ blade images are obtained. Then, motion correction^1^,^2^ is performed, and blade-combined images obtained by gridding and k-space sampling density compensation^1^,^19^. When SSB SENSE is used as a standalone method, these blade-combined images are the final ones.

### Multi-Step Joint-Blade SENSE (MJB SENSE)

**Figure 1:**
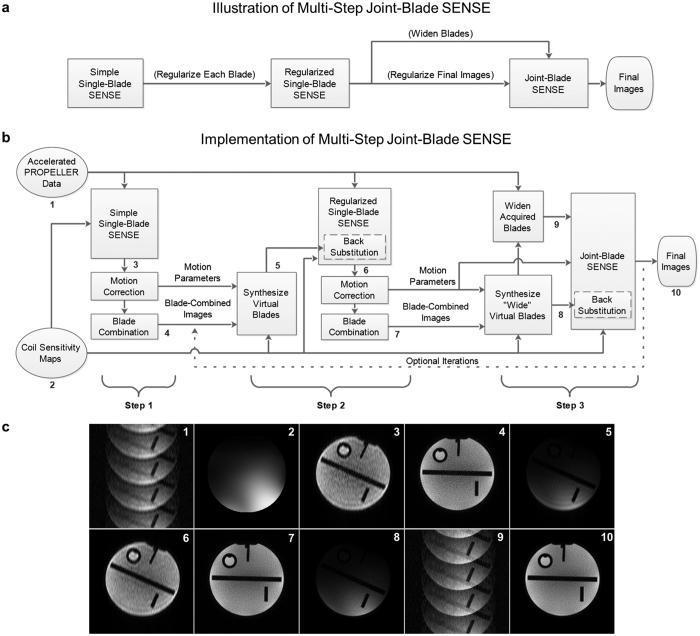
Illustration and implementation of the proposed multi-step joint-blade (MJB) SENSE reconstruction. (**a**) Illustration of MJB SENSE. Simple single-blade (SSB) SENSE is performed first to estimate blade-combined images, providing regularization to reconstruct each blade again in the next step. Then, relatively clean blade-combined images can be obtained, which are used to widen the acquired blades and form regularization for joint-blade SENSE. (**b**) The detailed implementation of MJB SENSE with motion correction. Motion parameters are initially estimated in Step 1 (SSB SENSE), updated in Step 2, and utilized in Step 3 (joint-blade SENSE). In Steps 2 and 3, the regularization (back-substitution) is performed through virtual blades that are resampled from the blade-combined images from the previous step. Blade widening is also performed through virtual blades. Optionally, the iteration (dashed line) is performed by feeding the reconstructed images from Step 3 to Step 2. (**c**) Illustration of the interim images in MJB SENSE as labeled in (**b**). image 1 – aliased blade images, image 2 – coil sensitivity maps, image 3 – SSB SENSE blade images, image 4 – SSB SENSE blade-combined images, image 5 – virtual blades, image 6 – the blade images reconstructed by Step 2, image 7 – the blade-combined images reconstructed by Step 2, image 8 – “wide” virtual blades, image 9 – widened blades, image 10 – MJB SENSE reconstructed images.

MJB SENSE is conceptually illustrated in Fig. 1a. Firstly, SSB SENSE is used to output the blade-combined images. Secondly, by utilizing SSB SENSE blade-combined images as regularization references, the blades are reconstructed again with reduced noise amplification. Thus, relatively clean blade-combined images can be also obtained. Thirdly, by resampling high-frequency data from these relatively clean blade-combined images, the acquired blades can be virtually widened so that their resolution inconsistency is eliminated. These widened blades can then be jointly reconstructed using SENSE to form the final images. Figure 1b shows the detailed implementation of MJB SENSE with motion correction. Blade-wise 2D motion parameters are initially provided by SSB SENSE, and updated in Step 2. The regularization is performed through virtual blades, which are resampled from blade-combined images to serve as the regularization references for the individual blades. Specifically, the regularization is implemented based on applying Jacobi method^20^,^21^ on the normal equations of equation (1). The details of these three steps are described as follows with interim images illustrated in Fig. 1c.

#### Step 1 – Simple Single-Blade SENSE

In this step, blade images (Fig. 1c image 3), motion parameters, and blade-combined images (Fig. 1c image 4) are estimated. The motion parameters and blade-combined images are passed to the next step as prior information.

#### Step 2 – Regularized Single-Blade SENSE

This step aims to reduce the noise in the blades, providing relatively clean blade-combined images for Step 3. For this purpose, virtual blades (Fig. 1c image 5) are resampled from the blade-combined images (Fig. 1c image 4). For example, to obtain virtual blade *i* in coil *k*, the blade-combined image is first shifted and rotated back to the uncorrected position of acquired blade *i*, as to undo motion correction. Then it is multiplied by the CSM of coil *k*, and Fourier transformed to k-space with the given blade width. These virtual blades resemble the blades reconstructed by SSB SENSE in terms of the phase encoding direction, blade width, and motion information, but they possess higher SNR.

The regularization is implemented by applying one iteration of Jacobi method^20^,^21^ on the normal equations of equation (1), i.e. 
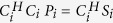
. For simplicity, we refer to this approach as “back-substitution”, since in essence, it updates the value of each unknown by treating the other unknowns as known. Without loss of generality, let *ρ*_*i*_ = *ρ*_1,*i*_ represent an arbitrary pixel in a blade. Equation (1) can be rewritten as:


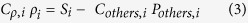


where *C*_*ρ*,*i*_ = *C*_1,*i*_ are the coil sensitivities on *ρ*_*i*_, and *P*_*others*,*i*_ = [*ρ*_2,*i*_ … *ρ*_*R*,*i*_]^*T*^ are the other pixels with coil sensitivities *C*_*others*,*i*_ = [*C*_2,*i*_ … *C*_*R*,*i*_]. Thus, the estimation of *ρ*_*i*_ that equation (2) yields can be related to the estimations of the other pixels aliased with it:


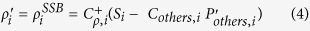


where 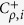 is simply 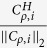 (conjugate normalized by *L*_*2*_-norm), and 
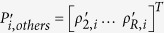
 are the estimations of the other pixels given by equation (2). Equation (4) shows the fact that, the estimation accuracy of 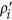 is closely related to the estimation accuracy of 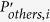. If an estimation containing less noise than 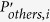 can be provided, 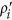 should be readily improved. Therefore, in Step 2, 
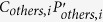
 in equation (4) are directly replaced with the corresponding values in the virtual blade images. Thus, Step 2 produces a new estimation of *ρ*_*i*_:


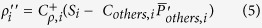


where
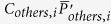
 corresponding to 
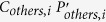
, are the sum of the other pixels in the virtual blade images. By comparing equation (5) and equation (4), it can be seen that 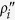 has higher SNR than 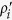, because 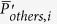
(generated from the blade-combined images) have substantially higher SNR than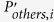 (obtained from a single blade without regularization). Thus, better estimations of the individual blades (Fig. 1c image 6) are obtained, and then motion parameters and blade-combined images (Fig. 1c image 7) are updated.

#### Step 3 – Joint-Blade SENSE

All acquired blades are first virtually widened: “wide” virtual blades (Fig. 1c image 8) are resampled with blade width *L*, from the Step 2 blade-combined images (Fig. 1c image 7). In other words, all wide virtual blades are generated to have the same resolution as the blade-combined images. Then, the virtual high-frequency data are patched from the wide virtual blades to the acquired blades. Thus, the resolution inconsistency among acquired blades is eliminated.

The widened acquired blades (Fig. 1c image 9) are jointly reconstructed with motion considered. First, despite the aliasing yet to be removed, all blades are co-registered by gridding each blade with rotation-corrected coordinates and translation-corrected phase^2^,^12^, according to motion parameters obtained in Step 2. Thus, the pixels in each blade finally have the same coordinates as in the final image. By combining SENSE equations from all blades, a joint-blade SENSE equation can be built as:


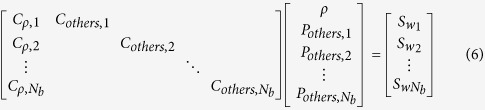


where *ρ* is the pixel being reconstructed, 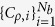 the coil sensitivities on *ρ* in each blade, 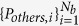 the other pixels aliased with *ρ* in each blade, 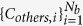 the coil sensitivities on 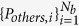, and 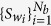 the signal intensities in the widened aliased blade images after co-registration. Equation (6) can be expressed more concisely as:


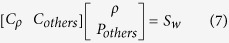


where *C*_*ρ*_, *P*_*others*_, and *S*_*w*_ are 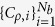, 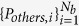, and 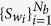 placed along column, respectively, and *C*_*others*_ is 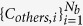 placed along diagonal. Equation (7) has moderate size and can be directly solved using pseudoinverse. However, we are only interested in *ρ* rather than *P*_*others*_. To speed up computation, back-substitution is used to replace *P*_*others*_ using the wide virtual blades. Back-substitution also reduces noise, preventing the estimation of *ρ* from being degraded by simultaneously estimating other pixels. Finally, Step 3 yields the estimation of *ρ*, i.e. the output of MJB SENSE, as:


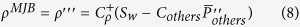


where 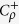 equals 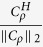, and 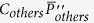 are the values corresponding to *C*_*others*_*P*_*others*_ in wide virtual blade images, which are also co-registered.

MJB SENSE described above is sufficient to substantially reduce noise amplification, yet additional iterations can be incorporated. For example, the iteration (dashed line in Fig. 1b) can be performed between Step 2 and Step 3, by feeding the reconstructed images from Step 3 to Step 2 to update the blade-combined images.

### Simulations

MJB SENSE was first evaluated using numerical simulations. Accelerated PROPELLER data sets were synthesized from one set of phantom images and one set of brain images. Both image sets were originally acquired using fast spin echo protocol at a 3 T MRI scanner (Philips Achieva). They contained 24 slices with matrix size 256 × 256 and coil number 8. The FOV of phantom and brain images were 26 and 24 cm, respectively. Sum-of-squares (SOS) images were first obtained by coil combination. Then CSMs were calculated with extrapolation^12^,^16^. These SOS images and CSMs were treated as the ground-truth for synthesizing PROPELLER data.

#### Noise Reduction Test

Both phantom and brain images were tested at acceleration factors of 4, 5, and 6. For each slice, non-accelerated PROPELLER data were first synthesized with blade number 16, blade width 10× acceleration factor (i.e. assuming ETL = 10 for accelerated data), and 256 data points per k-space line. The SOS images were multiplied by CSMs, then blade k-space was generated using image rotation and 2D FFT^22^. Here, the non-accelerated PROPELLER data were considered noise-free because they were purely simulated from the SOS images and CSMs. Any noise from the original MRI acquisition was simply treated as the texture of the object. Accelerated PROPELLER data were obtained by evenly discarding k-space lines in each blade.

Zero mean Gaussian noise was then numerically generated and added to the accelerated PROPELLER data to achieve the target signal-to-noise ratios (SNRs)^22^. SNRs of 10 and 20 were tested. The SNR was here defined as the ratio between the mean intensity of all slices and the standard deviation of the noise. Noise correlation was not considered. For simplicity, the gridding algorithm was based on image rotation and 2D FFT^22^. The reconstruction matrix size was 256 × 256. Monte Carlo g-factor maps were computed using pseudo multiple replica^23^ with 100 repetitions. The CSMs used in reconstruction were estimated using ESPIRiT^24^ from a simulated reference scan (48 × 48 data points with SNR = 20).

#### Motion Correction Test

Accelerated PROPELLER data with inter-blade in-plane rigid motion were synthesized using brain images. The motion parameters were generated with uniform random distribution in ±10 degrees for rotation and ±5 pixels for translation. To add motion into data, the SOS images were first shifted and rotated accordingly before CSM multiplication. Other steps for synthesizing data and adding noise were the same as described above without motion. Motion correction was based on the standard algorithm^2^.

### *In Vivo* Experiments

For demonstration, *in vivo* human brain data were acquired on a healthy volunteer using fast spin echo PROPELLER with acceleration factor R = 4, 5, and 6. Both T2-weighted (T2-w) and FLAIR T1-weighted (T1-w) PROPELLER data were acquired. The scan parameters for T2-w PROPELLER were TR/TE = 4000/120 ms, ETL = 30, blade number = 17 (for R = 6) or 20 (for R = 4 and 5), matrix size = 384 × 384, slice thickness = 4 mm, and FOV = 23 cm. The scan parameters for FLAIR T1-w PROPELLER were TR/TE/TI = 3000/43/1150 ms, ETL = 12, blade number = 15, matrix size = 236 × 236, slice thickness = 4 mm, and FOV = 23 cm. Note that the actual blade width is the product of ETL and acceleration factor. All scans were performed on a 3 T scanner (Philips Achieva) using an eight-channel head coil. Both motion-free and motion-corrupted data were acquired. For motion-corrupted data, the volunteer was asked to moderately rotate his head several times during the scan. All human experiments were approved by the Institutional Review Board of the University of Hong Kong and performed in accordance with the relevant guidelines and regulations. Informed consent was obtained from the subject. Note that inter-shot phase variation was found to be present in the data due to motion and hardware timing as previously reported^1^,^25^,^26^. To deal with this in MJB SENSE, the phase maps were estimated^1^ from the SSB SENSE blade images and multiplied to the virtual blades. The CSMs were calculated using ESPIRiT^24^ from a low-resolution reference scan.

In addition, the effect of adding iterations (between Steps 2 and 3) to MJB SENSE was briefly examined using the motion-corrupted T1-w data at R = 6.

## Results

### Noise Reduction

**Figure 2:**
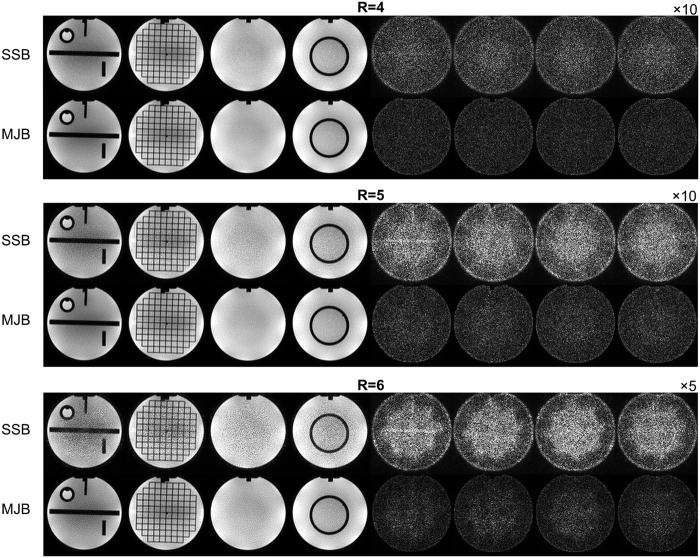
Reconstructed images and error maps in the noise reduction test using phantom images. MJB SENSE consistently resulted in substantially lower noise than SSB SENSE at all acceleration factors (R). The images shown were reconstructed from data with SNR = 20. The blade number was 16, ETL 10, and the number of coils 8. The error maps at R = 4, 5, and 6 are displayed at ×10, ×10, and ×5, respectively. All images were cropped to 200 × 200.

Figure 2 shows reconstructed images and error maps of four representative slices of the phantom images. In general, MJB SENSE resulted in much less noise than SSB SENSE. At R = 6, SSB SENSE led to very strong and apparent noise, which was substantially reduced by MJB SENSE.

**Figure 3:**
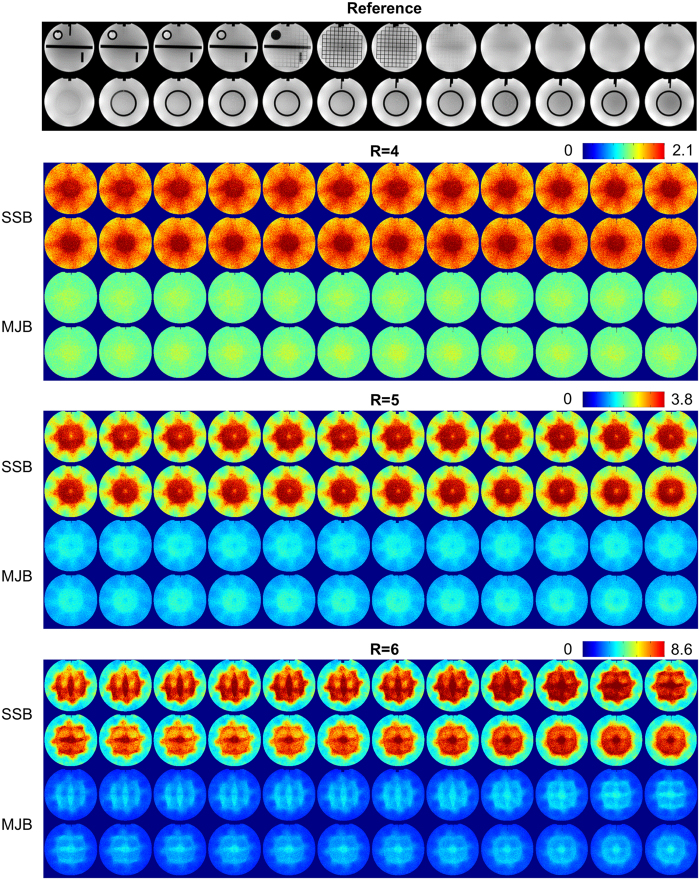
Monte Carlo g-factor maps of all 24 slices in the phantom images. These g-factor maps directly confirmed that MJB SENSE offered substantially lower noise amplification than SSB SENSE.

Figure 3 presents the Monte Carlo g-factor maps of all 24 slices using the two methods. These g-factor maps directly quantify the noise amplification. The overall g-factor spatial distributions were similar between the two methods. However, their quantitative values were obviously different. For example, the high g-factors near central FOV were substantially reduced in MJB SENSE.

**Figure 4:**
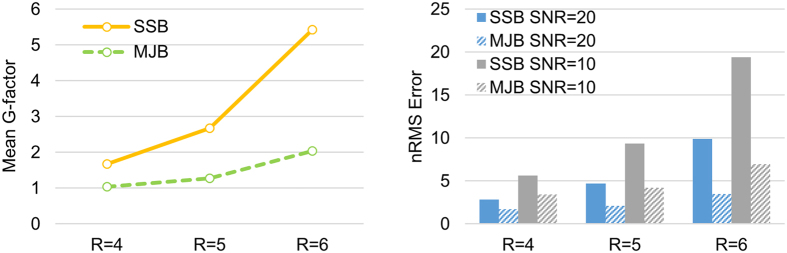
The mean g-factors and normalized root mean squares errors (nRMS) computed from all 24 slices of the reconstructed phantom images. The nRMS errors and g-factors were largely consistent, indicating that MJB SENSE greatly outperformed SSB SENSE in noise reduction. In general, MJB SENSE led to 30–60% reduction of the mean g-factor and nRMS error.

The mean g-factors and normalized root mean squares (nRMS) errors with acceleration factors are plotted in Fig. 4. The mean g-factors using SSB/MJB SENSE at R = 4, 5 and 6 were 1.68/1.04, 2.68/1.27, and 5.43/2.04, respectively, showing that MJB SENSE offered overall 30% to 60% less noise amplification than SSB SENSE. These results also indicated that MJB SENSE could increase acceleration factors, even with the small coil number of 8. In general, MJB SENSE led to 30% to 60% less nRMS error than SSB SENSE regardless of the noise levels. The nRMS error reduction was consistent with the g-factor reduction.

**Figure 5:**
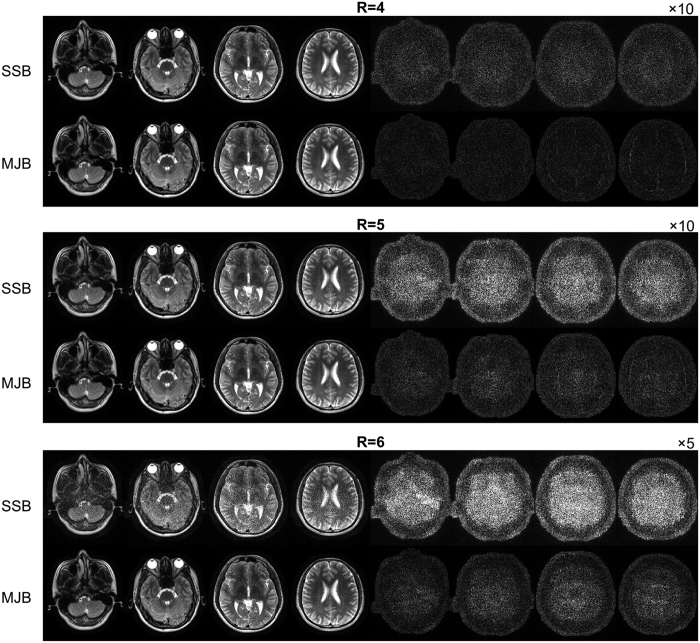
Reconstructed images and error maps in the noise reduction test using brain images. The results were similar to the phantom images shown in Fig. 2, again demonstrating substantial noise reduction using MJB SENSE. The fine structures inside brain were well reconstructed without apparent artifact. The images shown were reconstructed from data with SNR = 20. The error maps for R = 4, 5, and 6 are displayed at ×10, ×10, and ×5, respectively. The images were cropped to 220 × 256. The number of coils was eight.

Figure 5 shows the results of brain images with similar findings. Note that, at high acceleration factors, SSB SENSE images were extremely noisy whereas MJB images still largely recognizable.

### Motion Correction

**Figure 6:**
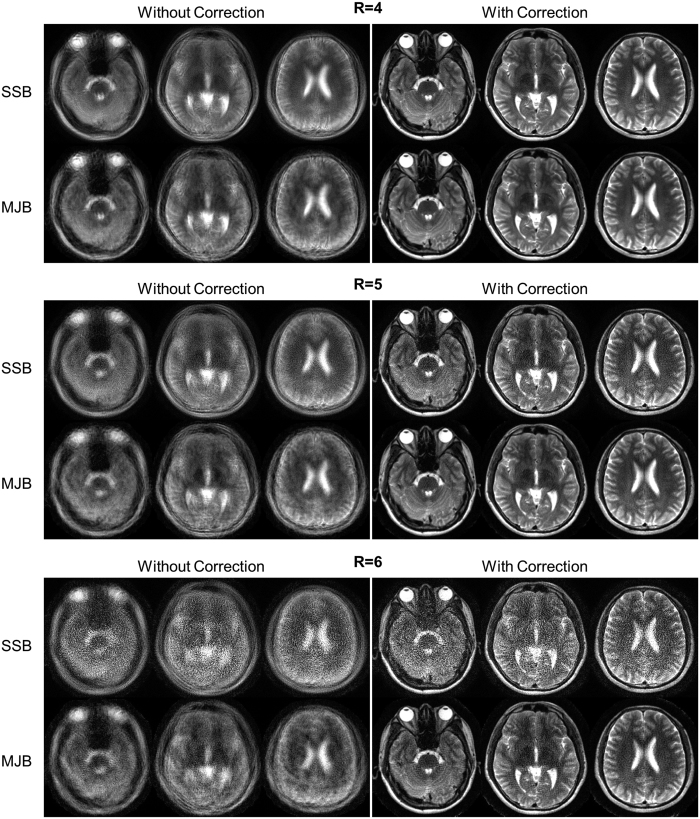
Motion correction results. Both methods could successfully remove motion artifact by motion correction. Even with high noise level at R = 6, SSB SENSE could still provide accurate motion estimation to ensure MJB SENSE being robust against motion. The number of coils was eight. The images were cropped to 200 × 220.

As shown in Fig. 6, both methods could successfully remove motion artifacts, demonstrating that the motion correction capability was largely preserved in MJB SENSE PROPELLER. Even when noise was high, as at R = 6, SSB SENSE could still provide good initial motion correction so that MJB SENSE remained robust against motion.

### *In Vivo* Experiments

**Figure 7:**
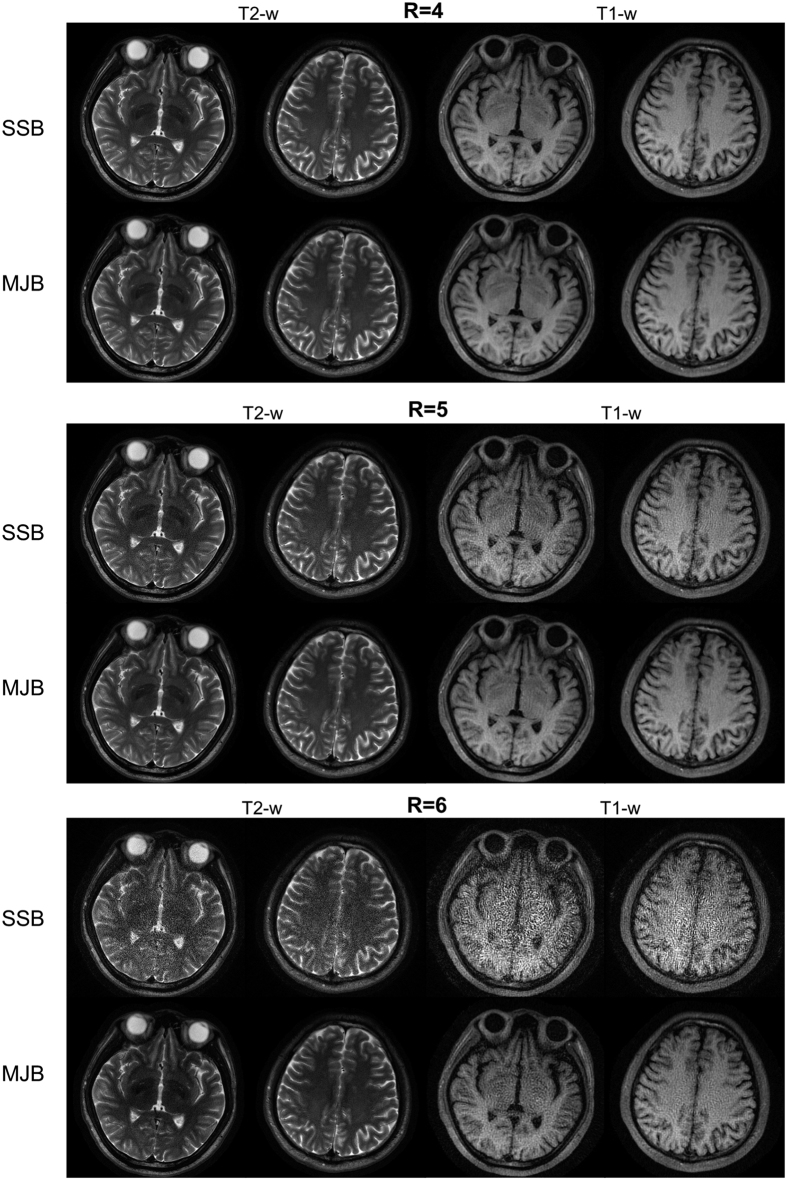
Images reconstructed from the *in vivo* brain data (without head motion) using PROPELLER with acceleration factor R = 4, 5 and 6 and an eight-channel coil. For both T1-weighted (T1-w) and T2-weighted (T2-w) imaging, MJB SENSE resulted in higher SNR than SSB SENSE. The improvement became increasingly apparent at higher acceleration factors. In particular, SSB SENSE failed to reconstruct meaningful images at acceleration factor of 6 whereas MJB SENSE still led to reasonably reconstructed images.

**Figure 8:**
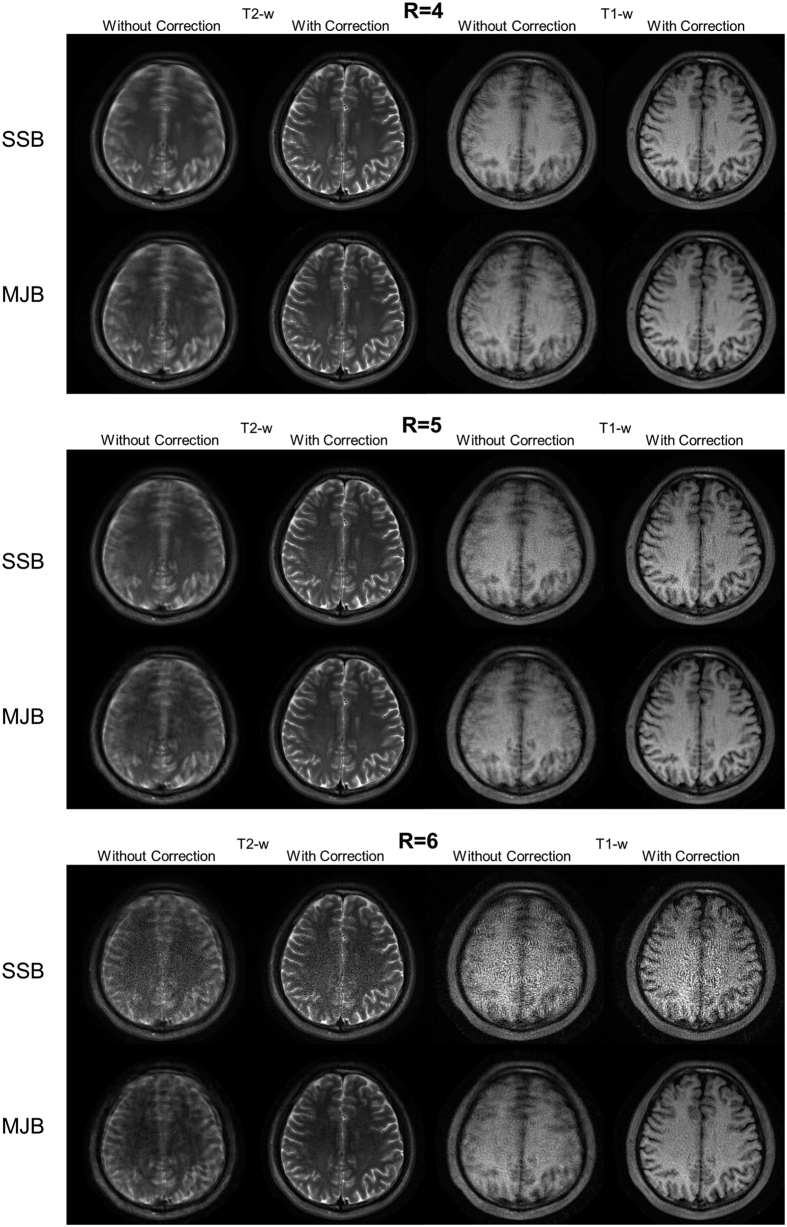
Similar to Fig. 7 but with head motions and subsequent motion correction. The level of motions was similar at different acceleration factors. The estimated rotations were approximately within ±6 degrees with standard deviation of ~3 degrees. The estimated translations (in left-right direction) were approximately within ±4 mm with standard deviation of ~1.5 mm. Despite the high acceleration factors and short echo train length of 12 for the T1-w data, MJB SENSE procedure shown in Fig. 1b could successfully correct the motion artifact.

As shown in Fig. 7, compared to SSB SENSE, MJB SENSE also substantially reduced noise amplification in the *in vivo* experiments, for both T2-w and T1-w imaging. Similar to the simulations above, MJB SENSE also achieved robust motion correction, as Fig. 8 shows. In particular, such motion correction was found to be effective in T1-w imaging with ETL as short as 12.

**Figure 9:**
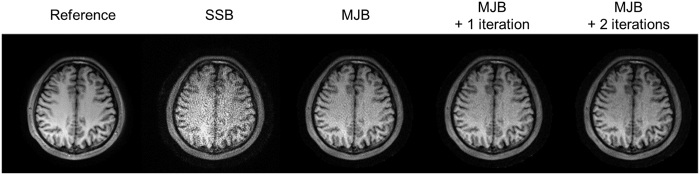
The effect of adding extra iterations to MJB SENSE. Here *in vivo* motion-corrupted data acquired at R = 6 were used for demonstration. The reference images were reconstructed from a fully sampled PROPELLER data set acquired before the accelerated one. The extra iterations further reduced noise, but the improvement was relatively minor.

As illustrated in Fig. 9, with the extra iterations, noise could be further reduced using MJB SENSE. However, this further improvement was minor when compared with that offered by MJB SENSE over SSB SENSE.

## Discussion

To reduce noise amplification, MJB SENSE exploits the complementary information from the rotatory blades in two ways: joint-blade SENSE and regularization using the blade-combined images.

First, joint-blade SENSE utilizes all blades in one equation, dramatically improving the conditioning of the inverse problem. Such concept has been employed in MUSE^25^ to improve SNR for Cartesian multi-shot imaging. Here, we overcome the difficulties associated with non-parallel nature of blades and presence of motion, and achieve joint-blade reconstruction without resorting to iterative methods^8^,^14^,^27^. As demonstrated in this study, once the resolution inconsistency is resolved, all blades can be gridded to a unified coordinate system and built into the same SENSE equation. Joint-blade SENSE is a pixel-wise operation due to the Cartesian nature of blades. In conjunction with back-substitution, it can be efficiently solved with good noise performance.

Second, the regularization using blade-combined images in Steps 2 and 3 is another form of joint-blade reconstruction in essence, because blade-combined images contain the information from all blades. Similar concept can be found in dynamic imaging^28^,^29^,^30^,^31^,^32^. Specifically, we use back-substitution, which is based on Jacobi method, to regularize the solutions of the SENSE equations (equations (5) and (8)), in a way similar to HYPR^28^,^30^, where the frame-combined image directly guides the back-projection process of individual frames. Such regularization is effective because the blade-combined images have much higher SNR than the single blade images (approximately by a factor of 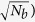.

Altogether, three factors above contribute to the noise reduction in MJB SENSE: back-substitution in Steps 2 and 3, and joint-blade SENSE itself. In fact, we also examined their respective effects. As shown by the nRMS errors (Table 1) and reconstructed images (Supplementary Figure S1) in individual steps, both back-substitution and joint-blade SENSE play important roles in noise reduction, yet their relative contributions vary from low to high accelerations. The contribution from joint-blade SENSE is typically dominant at low to moderate acceleration factors, whereas the contribution from back-substitution gradually grows with the increase of acceleration factors. Nevertheless, combining Step 2 and Step 3, as in the proposed MJB SENSE procedure, is generally better than repeating either of them. This is likely because Step 2 serves as a preprocessing step, and can effectively reduce the noise brought into Step 3 during the process of blade widening.

MJB SENSE can improve image quality by reducing noise at a given acceleration factor, or it can enable higher acceleration at a given noise tolerance. Apart from scan time reduction, this ability allows shortening echo train length, which particularly benefits T1-weighted PROPELLER, where relatively high acceleration is often needed for achieving short echo time and sufficiently wide blades simultaneously. For T2-weighted PROPELLER, shorter ETL also means less T2 blurring, less SAR, and more slices per TR.

SSB SENSE provides motion parameters for MJB SENSE. The two methods exhibit similar ability to correct either the simple simulated motion (Fig. 6), or more complex real motion (Fig. 8). Without proper motion correction, MJB SENSE may result in more artifacts than SSB SENSE. For example, Figs 6 and 8 show that, in the absence of motion correction, MJB SENSE could lead to more artifacts than SSB SENSE. This is not surprising because MJB SENSE needs correct motion parameters for virtual blade generation and joint-blade reconstruction. Nevertheless, for the relatively simple motion investigated in this study, SSB SENSE can robustly provide accurate motion parameters to guarantee that MJB SENSE is free from motion influence. For more complex motion, such as severe through-plane and intra-blade motion, other existing strategies such as weighting on blades according to motion severity^1^,^2^ may be incorporated in MJB SENSE. For example, as demonstrated in Supplementary Figure S2, through-plane motion artifact can be effectively suppressed by applying blade weighting in equation (8), whereas the weights can be obtained through correlation of the blades^1^.

Updating motion parameters in Step 2 may make little difference if they have been well estimated in Step 1. However, with more complex motion, the role of Step 2 may augment. Step 2 can improve motion estimation by reducing noise and artifacts in blades. It has the ability to output blade images, providing room for all possible strategies to be implemented for motion correction.

MJB SENSE can be combined with iterations to achieve extra noise reduction (Fig. 9). However, the improvement is minor, demonstrating that essence of MJB SENSE is not about iterations. Thus MJB SENSE is computationally efficient. It is fast, mainly involving least-squares estimation. Using Matlab codes on a desktop computer (4-core i7 CPU) without computational optimization, MJB SENSE took 12.7 seconds for each slice of the R = 5 T1-weighted data shown in Fig. 8, whereas SSB SENSE took 4.5 seconds. The time is expected to be dramatically reduced using C++ and better hardware, and online reconstruction should be highly feasible.

Self-calibration may be feasible in accelerated PROPELLER due to the oversampled central k-space, as demonstrated in our recent preliminary study^33^. However, the CSM obtained from self-calibration may be degraded in presence of severe motion, thus further studies are needed to investigate this issue.

Currently, the regularization in MJB SENSE is implemented based on one iteration of Jacobi method. When applied on normal equations, Jacobi method can be regarded as a special case of Landweber iterations^20^, which is known to have regularization effects^21^,^34^,^35^. Such implementation is simple and computationally efficient, even without the need of computing matrix inversion. Nevertheless, it is possible to use other regularization schemes such as Tikhonov^36^, total variation^37^,^38^,^39^, and low-rank constraints^40^,^41^.

Besides for motion correction, introducing weighting parameters in MJB SENSE may further improve SNR, stabilize iterations and reduce artifacts. Weighting may be introduced to MJB SENSE in various ways. For example, Jacobi over-relaxation method^20^ can be used for back-substitution, and virtual blade can be generated using a sliding window^31^ to weight more on specific blades.

Joint-blade SENSE can be performed twice after SSB SENSE for optimal performance at low acceleration factors (Table 1). By reconstructing the central k-space only, joint-blade SENSE may be feasible even without any blade-combined images. Also note that the most complementary blades should be the orthogonal ones^6^, and joint-blade SENSE may be reduced to two-blade SENSE for faster computation if needed. Nevertheless, these potential optimization and simplifications can be addressed in future studies.

Similar to SSB SENSE, SSB GRAPPA will likely suffer from noise amplification at high acceleration factors. Several existing GRAPPA PROPELLER methods^6^,^11^ utilize multiple blades for better GRAPPA kernel calibration, but they may not substantially improve SNR unless using more blades to interpolate the missing data. For joint-blade interpolation, pseudo-Cartesian GRAPPA^42^ deserves more investigation as a GRAPPA-based alternative to MJB SENSE. However, pseudo-Cartesian GRAPPA requires a number of kernels to deal with local k-space patterns. Designing these kernels is not straightforward, particularly if motion is considered. Iterative methods^8^,^13^,^27^,^43^,^44^,^45^ may be adapted to PROPELLER to achieve good noise performance, but they are much more computationally demanding in general. Moreover, almost all iterative methods have to be extensively modified to incorporate motion estimation and correction. Likely, any reconstruction methods suitable for accelerated PROPELLER need several steps to disentangle data and motion, similar to our proposed MJB SENSE.

Further optimization and evaluation of the proposed MJB SENSE PROPELLER reconstruction, including the motion correction ability, spatial resolution performance, and extension to other PROPELLER based techniques, are needed in the future. Due to the regularization and increased gridding operations, MJB SENSE may have slight resolution loss, which can be minimized by adjusting the relaxation parameters and choosing proper gridding algorithms. Currently, MJB SENSE has not considered some other inconsistencies among different blades, e.g., the T2/T2^*^ blurring effects and geometry distortions. In fast spin echo PROPELLER, these inconsistencies are insignificant as we demonstrated, yet they may manifest in other PROPELLER techniques^4^,^46^,^47^. For example, geometry distortion may cause pixel mismatch when applying MJB SENSE to PROPELLER EPI. Possible solutions include generating the virtual blade with higher weighting on its nearer blades, performing joint-blade SENSE separately on blade subsets that contain nearby blades only, and controlling the regularization strength with relaxation parameters^20^,^36^. In addition, the performance of the iterative MJB SENSE reconstruction as demonstrated in Fig. 9 has not been fully investigated in this study, which can be further explored in the future.

## Conclusion

In this study, a new reconstruction method, termed multi-step joint-blade SENSE (MJB SENSE), is proposed for accelerated PROPELLER MRI and demonstrated in both simulation and *in vivo* experiments. MJB SENSE PROPELLER exploits the sharable information existing among different blades, and incorporates regularization with the blade-combined images as the reference. Compared to conventional simple single-blade SENSE approach, MJB SENSE can substantially reduce noise amplification, and is particularly suited for high acceleration factors. MJB SENSE is robust even with short echo train length, offering more flexible contrasts. Furthermore, MJB SENSE preserves the PROPELLER motion correction capability and is computationally efficient.

## Additional Information

**How to cite this article:** Lyu, M. *et al*. A New Joint-Blade SENSE Reconstruction for Accelerated PROPELLER MRI. *Sci. Rep.* **7**, 42602; doi: 10.1038/srep42602 (2017).

**Publisher's note:** Springer Nature remains neutral with regard to jurisdictional claims in published maps and institutional affiliations.

## Supplementary Material

Supplementary Figures 1 and 2

## Figures and Tables

**Table 1:** The resulting nRMS errors by altering certain steps in MJB SENSE shown in Fig. 1b.

nRMS Error	R = 3			R = 4			R = 5			R = 6		
	After S1	After S2	After S3	After S1	After S2	After S3	After S1	After S2	After S3	After S1	After S2	After S3
SSB + RSB + JB (Standard MJB)	2.19	2.01	1.59	2.84	2.17	1.71	4.69	2.73	2.11	9.91	4.85	3.46
SSB + RSB + JB^^^ (No BS in S3)	2.19	2.01	1.60	2.84	2.17	1.80	4.69	2.73	2.40	9.91	4.85	4.34
SSB + RSB + RSB (S2 × 2, No JB)	2.19	2.01	2.00	2.84	2.17	2.04	4.69	2.73	2.33	9.91	4.85	3.57
SSB + JB + JB (S3 × 2, No RSB)	2.19	1.69	1.43	2.84	2.10	1.70	4.69	3.20	2.40	9.91	6.29	4.36

The results were obtained from simulated PROPELLER data with SNR = 20, ETL = 10 and blade number = 16. By comparing the rows, the contribution to noise reduction from different factors can be observed. From the first two rows, the contribution of back-substitution in Step 3 was found to be minor at R = 3 and 4, yet augmented at higher acceleration factors. From the last two rows, joint-blade SENSE was found to be the dominant factor for noise reduction at R = 3 and 4, whereas back-substitution in Step 2 contributed more at R = 5 and 6. Standard MJB SENSE was better than repeating either of Step 2 or 3 at R = 5 and 6, and was also near optimal at R = 3 and 4. These findings suggested that both back-substitution and joint-blade SENSE are important, and two are complementary in noise reduction. They also suggested that our proposed MJB SENSE is particularly optimal for high acceleration factors. At low acceleration factors, directly using joint-blade SENSE may be preferred. Notations: S1/2/3 - altered Step 1/2/3; SSB - simple single-blade SENSE; RSB - regularized single-blade SENSE; JB - joint-blade SENSE; BS - back-substitution; ^ - without back-substitution.
