# Supplementary material for: A New Joint-Blade SENSE Reconstruction for Accelerated PROPELLER MRI
Source: Sci Rep. 2017 Feb 16;7:42602. doi: 10.1038/srep42602 (PMC5311996; doi:10.1038/srep42602)
Supplement: Supplementary Figures 1 and 2 [file srep42602-s1.pdf]

# A New Joint-Blade SENSE Reconstruction for Accelerated PROPELLER MRI

Mengye Lyu, Yilong Liu, Victor B. Xie, Yanqiu Feng, Hua Guo, and Ed X. Wu

# Supplementary Information

## 1. Supplementary Figure S1.

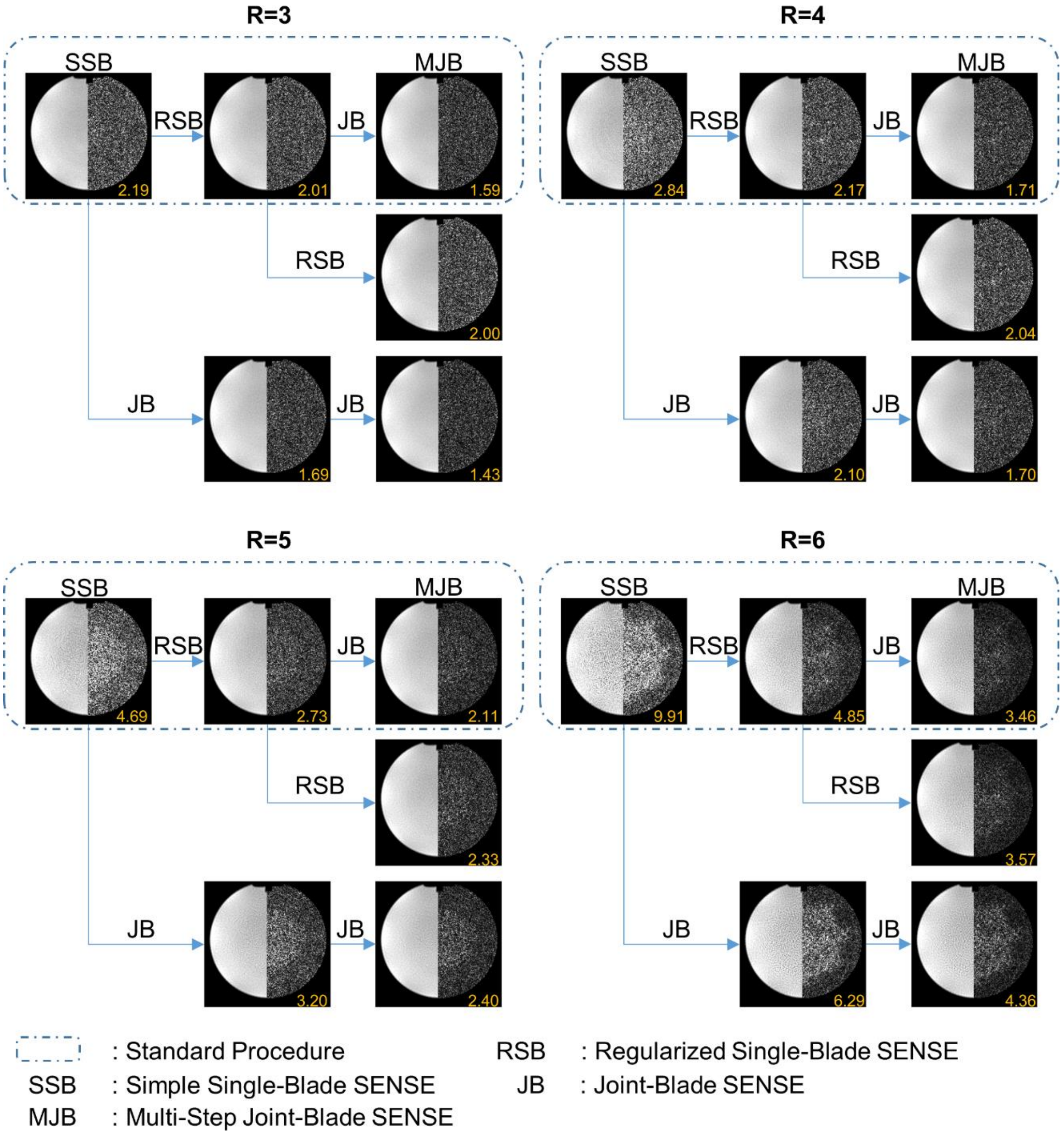


Supplementary Figure S1. The noise reduction effect in individual steps of MJB SENSE. Results are shown with standard procedure and possible variations as described in Table 1. The left half of each plotted image shows the reconstructed slice, while the right half shows the error map (displayed at × 15, 15, 10, and 5 at R=3, 4, 5, and 6, respectively). Normalized root mean square errors are in the lower right corners. As shown in the standard procedure, both Steps 2 and 3 in MJB SENSE reduced noise. By comparing the results of performing Steps 2 or 3 twice, joint-blade SENSE (Step 3) was found to be the dominant factor for noise reduction at R=3 and 4, whereas regularization using blade-combined images (Step 2) contributed more at R=5 and 6.

2. **Supplementary Figure S2.**

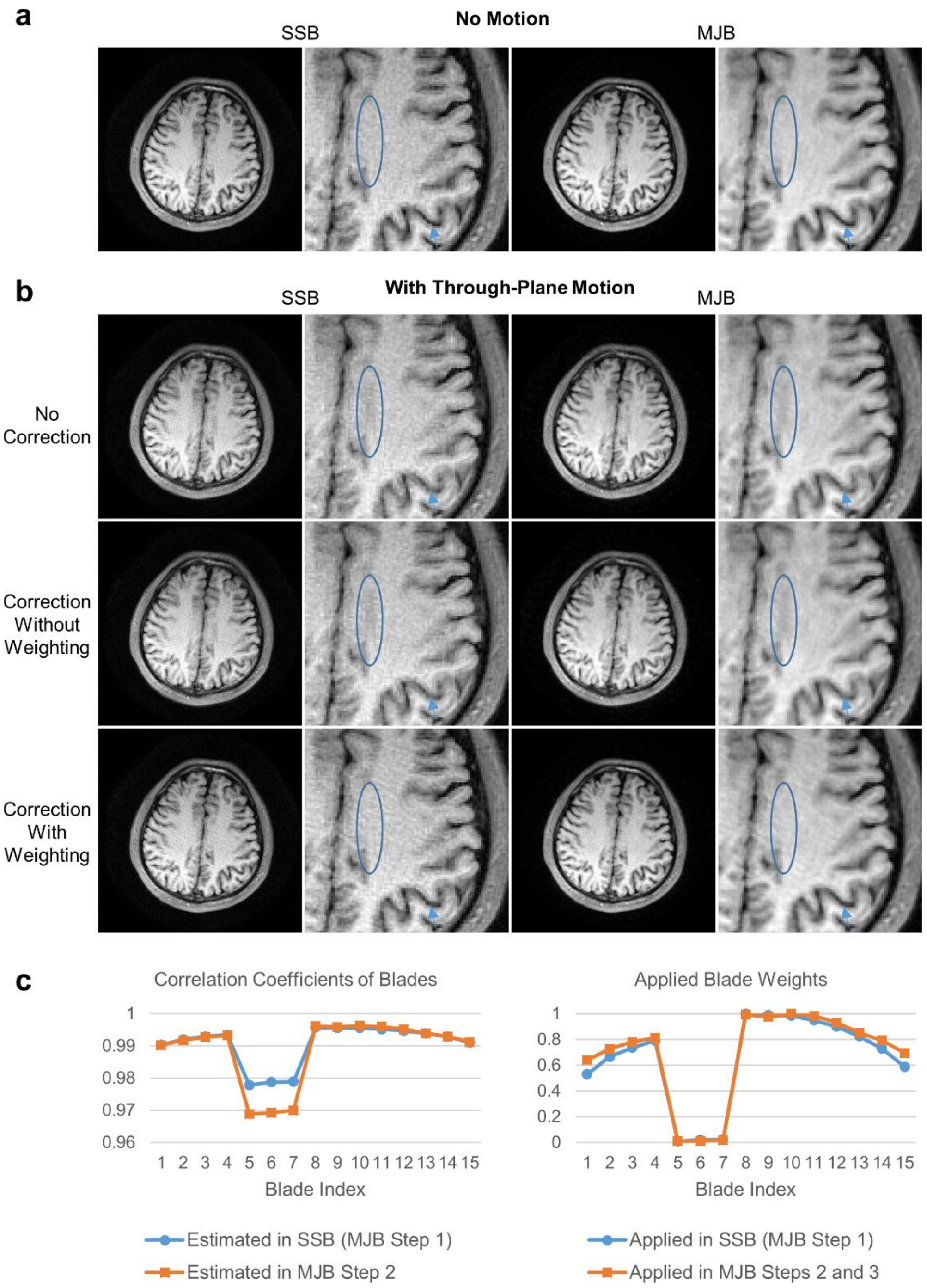


Supplementary Figure S2. Demonstration of introducing blade weighting in MJB SENSE to reduce through-plane motion artifact. (a) Acquired in vivo brain PROPELELR data at R=4 without intentional motion. (b) Hybrid simulation of through-plane motion. Three blades in (a) were replaced with the corresponding blades from another adjacent slice. Such through-plane motion could result in the dark artifact indicated by the circle and blurring indicated by the arrow. By applying blade weighting in equation (8) of MJB SENSE, the dark artifact and blurring were substantially reduced, while the SNR advantage of MJB SENSE was preserved. (c) Determination of the blade weights in (b). First, the correlation coefficient of each blade was calculated with regard to an averaged blade image. Then the correlation coefficients were mapped to blade weights using method described in Ref. 1. Thus, the three blades (blade #5, #6, and #7) containing through-plane motion were identified and given very small weights.

### 3. Equation (8) with blade weights

When applying blade weights, equation (8) becomes

$$\rho^{MJB} = \rho''' = \left(WC_{\rho}\right)^{+} W(S_w - C_{others}\bar{P}''_{others}),$$

where $W$ is a $N_c \times N_b$ – by – $N_c \times N_b$ diagonal matrix, with each blade weight applied to all coils as the diagonal elements.
